# Characterizing the tumor suppressor activity of FLCN in Birt-Hogg-Dubé syndrome through transcriptiomic and proteomic analysis

**DOI:** 10.21203/rs.3.rs-4510670/v1

**Published:** 2024-06-26

**Authors:** Andrew Tee, Rachel-Ann Jones, Elaine A. Dunlop, Jesse Champion, Peter Doubleday, Tijs Claessens, Zahra Jalali, Sara Seifan, Iain Perry, Peter Giles, Oliver Harrison, Barry Coull, Arjan Houweling, Arnim Pause, Bryan Ballif

**Affiliations:** Cardiff University; Cardiff University; Cardiff University; Cardiff University; University of Cardiff; University of Cardiff; University of Cardiff; University of Cardiff; McGill University; McGill University; McGill University; McGill University; McGill University; McGill University

## Abstract

Birt-Hogg-Dubé (BHD) syndrome patients are uniquely susceptible to all renal tumour subtypes. The underlying mechanism of carcinogenesis is unclear. To study cancer development in BHD, we used human proximal kidney (HK2) cells and found that long-term folliculin *(FLCN)* knockdown was required to increase their tumorigenic potential, forming larger spheroids in non-adherent conditions. Transcriptomic and proteomic analysis uncovered links between FLCN, cell cycle control and the DNA damage response (DDR) machinery. HK2 cells lacking *FLCN* had an altered transcriptome profile with cell cycle control gene enrichment. G_1_/S cell cycle checkpoint signaling was compromised with heightened protein levels of cyclin D1 (CCND1) and hyperphosphorylation of retinoblastoma 1 (RB1). A FLCN interactome screen uncovered FLCN binding to DNA-dependent protein kinase (DNA-PK). This novel interaction was reversed in an irradiation-responsive manner. Knockdown of FLCN in HK2 cells caused a marked elevation of γH2AX and RB1 phosphorylation. Both CCND1 and RB1 phosphorylation remained raised during DNA damage, showing an association with defective cell cycle control with *FLCN* knockdown. Furthermore, Flcn-knockdown *C. elegans* were defective in cell cycle arrest by DNA damage. This work implicates that long-term *FLCN* loss and associated cell cycle defects in BHD patients could contribute to their increased risk of cancer.

## Introduction

Birt-Hogg-Dubé syndrome (BHD, OMIM # 135150) is an autosomal dominant condition, clinically characterized by fibrofolliculomas, pneumothorax associated with multiple lung cysts and early-onset multiple renal cell cancers (RCC) [1, 2]. BHD is caused by inactivating germline variants in the *FLCN* (folliculin) gene [2]. Lifetime risk of RCC in BHD has been estimated to be around 15%. BHD patient renal tumors may show a variety of histological subtypes including mixed forms [3]. Most BHD renal cell cancers grow slowly, but metastatic disease may present as the first manifestation of the disease. Surveillance for early detection of renal lesions and surgical treatment of localized RCC is based on insight into the biological behavior of these tumors. Many BHD patients develop multiple and bilateral renal tumors and nephron sparing surgery is therefore essential [2]. There is evidence of bi-allelic inactivation of *FLCN* in many BHD RCC. Targeted treatments for such tumors are not currently available due to a lack of basic understanding of the key pathways involved in tumour growth.

While major advances have been made, the underlying mechanism of how BHD tumors develop over time remains unclear. Current evidence indicates that the core function of FLCN is in lysosomal nutrient sensing and autophagy control, through FLCN’s control of the Rag GTPases and thus mTORC1 phosphorylation of TFE3 and TFEB [4, 5, 6, 7]. Further crosstalk with lysosomal pathways likely come via both AMPK and ULK1-mediated phosphorylation of FLCN [8] and TFEB/TFE3 phosphorylation via AMPK [9]. As a direct or indirect result of lysosomal dysfunction, loss of *FLCN* can cause abnormal ciliogenesis [10] and defects in metabolic homeostasis [11, 12]. FLCN has also been linked to a variety of fundamental cellular processes including cell adhesion through plakophilin-4 (PKP4) [13, 14] and cell growth and division through rRNA synthesis [15], cyclin D1 (CCND1) and marked changes in gene expression [16]. RagC-independent functions of FLCN are also becoming apparent in recent research, where FLCN was found to be a Rab7A GTPase activating protein involved in endocytic trafficking of epidermal growth factor receptor [17]. A related novel hereditary disorder was linked to *PR/SET Domain 10(PRDM10)* that predisposed families to skin and mucosal lesions, lipomatosis and renal cell carcinomas [18, 19]. The *PRDM10* mutation that co-segregated with disease was found to be defective at binding to *FLCN* promoter regions and consequently caused a reduction in FLCN expression [18].

Although FLCN’s involvement in cell growth control is better understood, it is still not clear how dysregulation of this pathway following FLCN loss could predispose patients to multiple RCC subtypes with different mutational signatures [20]. Therefore, we wanted to further study the tumorigenic mechanisms underlying *FLCN* loss. We observed increased tumorigenic properties after long-term knockdown of FLCNin HK2 cells (after they were grown for a year in continuous tissue culture), while short-term *FLCN* knockdown showed little capacity to transform these cells. We examined the transcriptome profile of these cells to reveal enrichment of genes linked to cell cycle control, a tumorigenic feature that became more prominent with long-term loss of *FLCN*. To better understand the function of FLCN, we then carried out proteomic analysis of FLCN interactors, which also mapped to multiple cell cycle regulators and included components of the DNA damage machinery. Our findings suggest that alterations in cell cycle control upon *FLCN* loss would enhance cancer progression in BHD.

## Materials and Methods

### Cell culture

Unless stated otherwise, lab reagents were purchased from Merck Millipore (Burlington, Massachusetts, USA). HEK293 were purchased from American Type Culture Collection (Manassas, Virginia, USA). Cell maintanence and the generation of stable *FLCN* knockdown HK2 cells are described [10]. Short and long-term knockdown refers to < 30 and >105 passages after clonal selection, respectively. Lipofectamine 2000 transfection was carried out according to the manufacturer’s protocol (Life Technologies, Paisley, UK). Irradiation (IR) was carried out using Gammacell 1000 Elite (Nordion Gamma Technologies, Abingdon, UK). Cells were checked with Venor^™^ GeM Advance Mycoplasma Detection Kit (Minerva Biolabs, Berlin, Germany) as per manufacturer’s guidelines and were mycoplasma negative. Tumor spheroid growth assays in soft agar are previously described [21]. For spheroid protein analysis, 12,500 cells were allowed to self-aggregate in 1.5% agarose coated wells. Forty-eight spheroids per cell line were lysed in 1x sample buffer (62.8 mM Tris, 10% (v/v) glycerol, 2% (w/v) SDS, 0.1% (w/v) bromophenol blue, with 50 mM dithiothreitol (DTT) added just before use), sonicated and boiled prior to blotting. Cell lysis and western blot analysis are described [22].

### Antibodies

Unless specified elsewhere, antibodies were purchased from Cell Signaling Technologies (Danvers, MA, USA). Phospho-specific antibodies include: p-H2AX Ser139 (#9718P (20E3)), p-TP53 Ser15 (#9286P (16G8)), p-RB1 Ser780 (D59B7), p-AMPK Thr172 (#2531) p-ACC Ser79 (#3661) and p-DNA-PK Ser2056 (Ab124918 from Abcam Plc, Cambridge, UK). Pan antibodies include: β-actin (#84573 (D6A8)), CCND1 (#2922), CDKN1A (12D1), SQSTM1 (#5114), TP53 (DO-1 from Bethyl Laboratories Ltd., Montgomery, TX, USA), and from Merck Millipore both GST (#DAM1411332), ATM (#2873 (D2E2)), ATR (#2790), and DNA-PKcs (#D00148436), HA (#1186742300 from Roche Products Limited, Welwyn Garden City, UK). Custom made N-terminal anti-FLCN is previously described [10].

### GST pull down and co-immunoprecipitation assays

HEK293 cells were transfected with either GST-FLCN in pDEST27 backbone (Life Technologies, 11812013) or pcDNA3 empty vector. 150 μL of lysate was incubated with pre-blocked glutathione-Sepharose 4B beads (GE Healthcare, Chalfont St. Giles, UK) at 4°C in a rotary shaker for 3 h. Beads were washed 5 times in BHD lysis buffer [8] with 300 mM NaCl for DNA-PKcs (250 mM for all other proteins). Bound proteins were eluted using 10 mM glutathione in elution buffer (20 mM HEPES (pH 8), 200 mM NaCl, 5 mM MgCl_2_) to avoid elution and subsequent detection of proteins that bound non-specifically to the beads. Elutent was mixed with NuPAGE LDS sample buffer containing 100 mM DTT. For co-immunoprecipitation, HEK293 cells were transfected with either HA-FLCN wild type, HA-FLCN Y463X or HA-FLCN H429X in pcDNA3.1 vector or pcDNA3.1 empty vector (described in [8]). Lysates were pre-cleared with unblocked protein G-Sepharose beads (GE Healthcare) for 1 h at 4°C, then centrifuged for 3 min, 3 000 rpm at 4°C to remove beads. Lysates were then incubated with anti-HA antibodies for 2 h at 4°C in a rotary shaker before adding BSA-blocked protein G-Sepharose beads for 2 h. Beads were washed then proteins were dissociated with sample buffer containing 25 mM DTT at 70°C for 10 min.

#### Mass spectrometry sample preparation, sequencing, and FLCN protein-protein interaction analysis.

Proteins were identified from gel fragments by liquid chromatography-tandem mass spectrometry (LC-MS/MS), as previously described [23]. Identified proteins were filtered by removal of keratins. Proteins with 2 or more unique peptides identified were analysed using the Database for Annotation, Visualization and Integrated Discovery (DAVID) Functional Annotation Clustering [24, 25]. Known FLCN interactors were screened manually and using the GO terms: (i) protein folding GO:0006457, (ii) metabolic processes GO:0031323, (iii) cell cycle GO:0007049 and (iv) response to DNA damage: GO:0006974.

### Statistical analysis

Unless stated otherwise, after determining normal (Gaussian) distribution (Shapiro-Wilk test), ordinary one-way ANOVA with Tukey’s multiple comparisons was carried out using Prism GraphPad; * p< 0.05, ** p < 0.01, *** p< 0.001, **** p < 0.0001, and not significant (ns). Data are all presented as mean ± SEM. Experiments were repeated 3 times, unless stated otherwise.

Other methods are found in supplementary.

## Results

### Long-term knockdown of FLCN increases tumorigenesis

**Figure 1:**
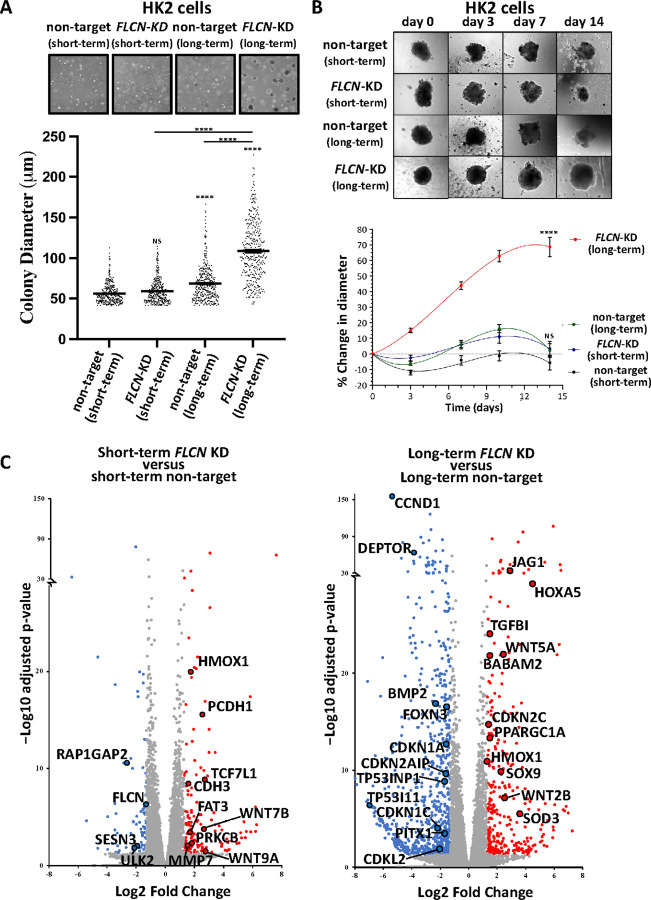
Long-term knockdown of FLCN increases tumorigenesis. **(A)** HK2 cells with and without short-term or long-term *FLCN* shRNA knockdown (compared to non-target shRNA) were grown in soft agar for 21 days. Colony diameter was measured using ImageJ. (v1.53t), and a representative image of tumors is shown and distribution of tumors graphed. n=360 per condition over 6 biological samples. Each data point is a single tumor. Stats: distributed data was not normal (D’Agostino & Pearson test) so was analysed using nonparametric Kruskai-Wallis ANOVA with Dunn’s multiple comparisons. (**B**) These HK2 cells were also plated in non-adherent conditions and imaged over 14 days, and % change in diameter graphed over time (*n*=42). RNA sequencing of HK2 cells with either short- or long-term non-target or *FLCN* shRNA knockdown was carried out (*n*=3). (**C**) Differential gene expression was compared in short-term FLCN versus non-target shRNA knockdown and long-term FLCN versus non-target shRNA knockdown HK2 cells. Volcano plots are shown with the following thresholds: ≤ & ≥ 2.5 fold change, padj < 0.05 with false rate discovery (FDR) correction applied. Relative gene expression for selected genes are shown.

To mimic long-term haploinsufficiency of FLCNin kidney cells within BHD patients, we continuously grew HK2 cells, a human proximal tubule cell line, with and without *FLCN* knockdown for one year in tissue culture to allow for long-term effects of *FLCN* knockdown to become apparent. To compare short-term effects, cells were grown for < 2 months after *FLCN* knockdown. To investigate whether long-term *FLCN*-deficiency increased tumorigenicity, we compared the growth of these *FLCN* knockdown HK2 cells in soft agar (Fig. 1A). Short-term *FLCN* knockdown did not alter colony formation, while long-term *FLCN* knockdown led to much larger colonies, with a mean diameter >100 μm. Colony diameters within the long-term *FLCN* knockdown population were more divergent, showing heterogeneity in colony formation.

To further assess anchorage-independent growth, these HK2 cells were grown as self-aggregated spheroids (Fig. 1B). We observed enhanced tumorigenity after long-term *FLCN* knockdown, when compared to short-term knockdown. We next analyzed spheroid growth of additional non-target and *FLCN* shRNA knockdown clones (Supplementary Fig. 1) that supports our finding that long-term *FLCN* knockdown cells showed the greatest increase in spheroid diameter. This data reveals that long-term *FLCN* knockdown has higher capacity to promote tumorigenicity, when compared to short-term knockdown.

#### FLCN knockdown dramatically altered the transcriptome profile over time, showing gene enrichment of cell cycle genes.

**Figure 2:**
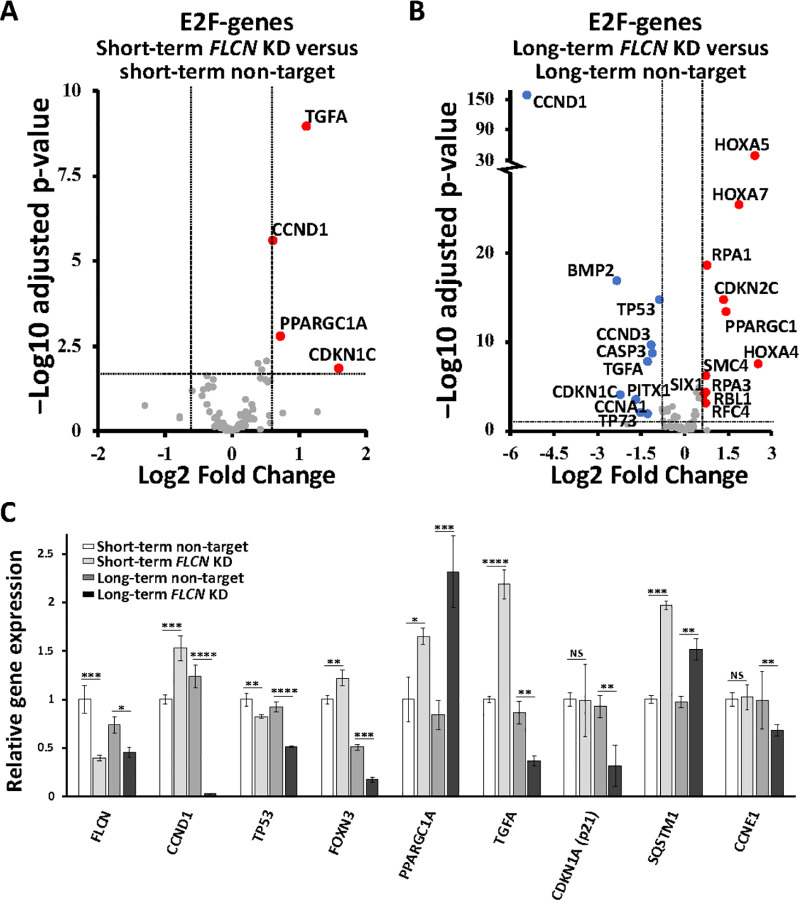
Differentially expressed genes linked to E2F and known FLCN regulatory genes after FLCN knockdown. Differential gene expression of E2F-genes was compared in (**A**) short-term *FLCNversus* non-target shRNA knockdown and (**B**) long-term *FLCN* versus non-target shRNA knockdown HK2 cells, after RNA sequencing. Volcano plots are shown with the following thresholds: ≤ & ≥ 2.5 fold change, padj < 0.05 with false rate discovery (FDR) correction applied. (**C**) FLCN linked genes from this RNA sequencing experiment are graphed, and include FLCN, CCND1, TP53, PPARGC1A, TGFA, CDKN1A, SQSTM1 and CCNE1.

Transcriptional changes between non-target and *FLCN* knockdown in HK2 cells were investigated. A PCA and Euclidean cluster dendrogram plot of DESeq2 normalized samples showed that experimental replicates cluster tightly in distinct groups. The most significant factor of interest (PC1) separates the long-term *FLCN* knockdown group from all other conditions (Supplementary Fig. 2A-B). While short-term *FLCN* knockdown altered expression of 216 genes, this strikingly rose to 1063 genes after long-term *FLCN* knockdown (Fig. 1C, ≥ 2.5 fold change up or down, padj < 0.05 with false rate discovery (FDR) correction applied). A WNT signaling signature was evident after both short- and long-term *FLCN* knockdown. Differentially expressed genes from long-term *FLCN* knockdown showed greatest enrichment in ‘Aberrant regulation of mitotic G_1_/S transition in cancer due to RB1 defects’, Reactome: R-HSA-9659787 (> 100 fold enrichment and 7.65E-04 FDR correction applied). Therefore, we investigated RB1/E2F-regulated genes involved in S-phase entry. When comparing short-term *FLCN* knockdown to its wild-type control (Fig. 2A), *TGFA, CCND1, PPARGC1A* (also known as *PGC1a)* and *CDKNICwere* upregulated with *FLCN* knockdown. After long-term *FLCN* knockdown, many more genes were differentially expressed. Three *HOX* genes showed the greatest fold increase, while *TP53, BMP2, TGFA* and *CCND1* were downregulated (Fig. 2B). Changes of E2F-regulated genes: *CCND1, TP53, PPARGC1A, TGFA, c-Jun, RPA1* and *RBL1* [26] are shown in Fig. 2C, along with *p21 (CDKN1A)* and *FOXN3*that can arrest cells at the G_1_ checkpoint [27, 28]. mRNA expression of *CCND1, TP53, FOXN3, p21 (CDKN1A), TGFA* and *CCNE1* were downregulated after long-term *FLCN* knockdown. Supporting these observed alterations in cell cycle regulatory components, *FLCN* knockdown cells showed small but significant changes in 2N (G_0_/G_1_) and 4N (G_2_/M) by flow cytometry, when compared to wild-type (Supplementary Fig. 2C).

**Figure 3:**
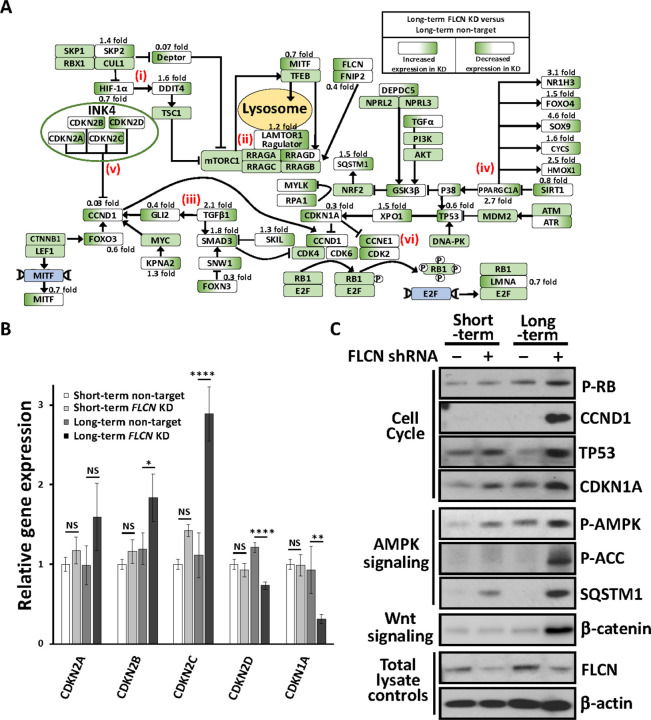
Differentially expressed genes and their associated signaling pathways. (**A**) Gene expression comparing long-term *FLCN* shRNA versus non-target shRNA knockdown in HK2 cells, after RNA sequencing. Each gene is depicted in a signaling flow diagram. Known signaling functions of FLCN include: (i) Enhanced HIF-1a activity, as shown by increased HIF-1a target gene expression, *SLC2A2* and *REDD1*. Signaling feedback mechanism to reduce HIF-1a, through reduced HIF-1a expression and upregulation of *SKP2,* a HIF-1a inhibitor. (ii) Upregulation of mTORC1 through enhanced expression of *LAMTOR1* and a marked reduction in *DEPTORexpression,* an mTORC1 inhibitor. (iii) Upregulation of TGFb/SMAD3 signaling upon knockdown of *FLCN*. SMAD3 is an inhibitor of CCND1-CDK4/CDK6 complexes. (iv) PGC1a and downstream regulated genes, *NR1H3, FOXO4, SOX9, CYCS, HMOX1* are markedly upregulated upon *FLCN* knockdown. (v) The INK4 family members that inhibit CCND1, *CDKN2A-C,* are increased while *CDKN2D* is reduced. (vi) There is reduced expression of *CDKN1A* that inhibits both CCND1 and CCNE1 activity. This demonstrates a transcriptional feedback mechanism to enhance CDK4/6 and CDK2 activity. Further differences were observed with enhanced CDK6expression and lower expression of *CCND1* and *CCNE1*. (**B**) Relative gene expression of cyclin dependent kinase inhibitors across the different HK2 cell lines was calculated, (*n*=3). (**C**) Protein lysates from spheroids generated from HK2 cells with and without short- or long-term *FLCN* shRNA knockdown (non-target shRNA used as a control) were probed for phosphorylated RB1, CCND1, TP53, CDKN1A, phosphorylated AMPK and ACC, SQSTM1, b-catenin, FLCN, and b-actin as a loading control (*n*=3).

To obtain a sense of this dysregulation at a pathway level, we assessed the signaling flow from *FLCN* knockdown to E2F changes (Fig. 3A). Upon long-term *FLCN* knockdown we observe marked gene expression alterations in multiple pathways that have been previously linked to BHD: (i) upregulation of *HIF1A* [12] towards gene targets, SLC2A2 (11.2 fold) and DDIT4 (1.6 fold). (ii) Dramatic reduction of DEPTOR (by 93%), the negative regulator of mechanistic target of rapamycin complex 1 (mTORC1). (iii) Enhanced expression of TGFp (2.1 fold increase) and SMAD3 (1.8 fold increase), supporting elevated TGFp-SMAD signaling [30]. (iv) 20% reduction in SIRT1 expression (negative regulator of PPARGC1A) and a marked 2.7 fold increase in *PPARGC1A* expression, and subsequent increase of PPARGC1A-regulated genes: *NR1H3, SOX9,* and *HMOX1* (> 2.5 fold change and < 0.0001 adjusted pvalue). This observation supports previously published work where *PPARGC1A* was observed to drive mitochondrial biogenesis and metabolic transformation in BHD cell models [11]. More than half of the G_1_/S regulatory genes show dysregulation following *FLCN* knockdown, with notable changes to (v) INK4 family members that negatively regulate CCND1 (graphed in Fig. 3B, CDKN2A-D) and (vi) cell cycle regulators that control RB1 phosphorylation and E2F activation. We also see evidence of *FLCN* knockdown cells employing feedback mechanisms to reduce G_1_/S regulatory component expression. For example, although initially upregulated upon *FLCN* knockdown (Fig. 2C and Supplementary Fig. 4), *CCND1* mRNA was strikingly downregulated by 97% after long-term *FLCN* knockdown (Fig. 2C and 3A). As there is a bidirectional relationship between metabolism and cell cycle progression [29], we speculate that the altered metabolism observed in FLCN-deficient cells [11, 12] could underlie the defects in E2F-regulated cell cycle gene expression that becomes more marked and pro-tumorigenic over time.

To assess whether alterations in spheroid growth correlated with the observed alterations in cell cycle pathway dysregulation, we generated lysates from the spheroids grown in Fig. 1B and analyzed a panel of cell cycle proteins. We found that spheroids from long-term *FLCN* knockdown had elevated expression of CCND1 protein and RB1 phosphorylation (Fig. 3C). These alterations after long-term *FLCN* knockdown could correlate with their increased growth properties. As negative regulators of cyclin:cyclin dependent kinase (CDK) complexes, we analyzed TP53 and cyclin-dependent kinase inhibitor 1A (CDKN1A, known as p21/WAF1). We found them highly expressed following *FLCN* knockdown, and in both cases the levels were higher after long-term knockdown. This contrasts with the RNA data, where *CCND1, TP53* and *CDKN1A* mRNA expression were all downregulated with long-term *FLCN* knockdown (Fig. 2C). Long-term *FLCN* knockdown also elevated the expression of β-catenin showing increased WNT signaling, in line with our RNA sequencing findings (Fig. 1C). Sequestosome 1 (SQSTM1, known as p62) mRNA was also increased by 1.5 fold (Fig. 2C) and protein expression was markedly enhanced (Fig. 3C). AMPK and Acetyl-CoA carboxylase (ACC) phosphorylation was enhanced following *FLCN* knockdown that was more evident after long-term knockdown (Fig. 3C). High levels of SQSTM1 and AMPK activation is consistent with metabolic alterations and energy stress, as previously reported in BHD models [11].

### FLCN interacts with components of the cell cycle and DDR

**Figure 4:**
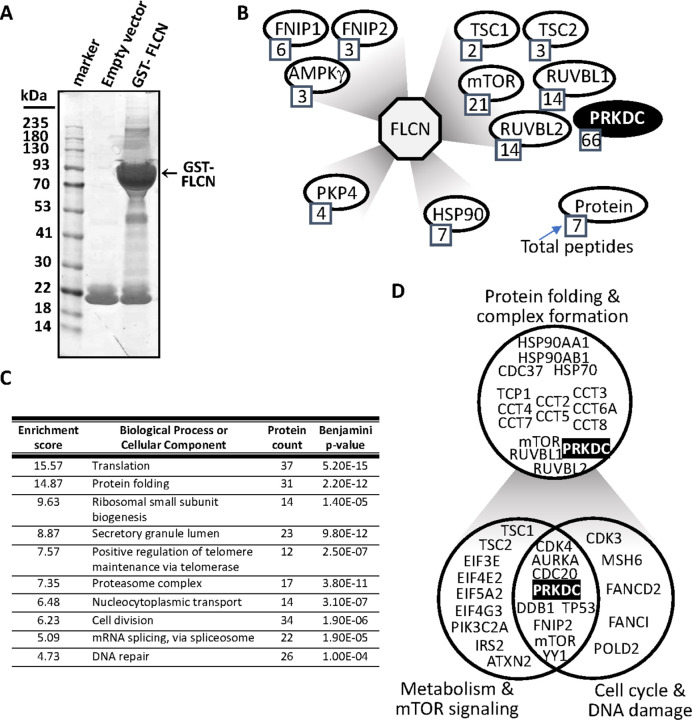
FLCN has an extensive PPI network linked to cell cycle and DNA damage. **(A)** GST-FLCN was overexpressed in HEK293 cells, purified and interacting protein separated by SDS-PAGE and stained with colloidal blue. The gel was sectioned for mass spectrometry analysis. **(B)** FLCN protein interaction network is represented showing known FLCN interactions and includes PRKDC. Total peptides of protein sequences identified after mass spectrometry is indicated. (**C**) FLCN binding proteins identified by mass spectrometry was analyzed by DAVID, and the top ten enriched scored biological processes or cellular component are presented. (**D**) FLCN binding proteins are grouped together in protein folding and complex formation, metabolism and mTOR signaling, and cell cycle and DNA damage.

To further define FLCN as a tumor suppressor, mass spectrometry of GST-tagged FLCN-interacting partners was performed (Fig. 4A). This uncovered 603 potential FLCN-binding proteins, and successful enrichment for FLCN binding proteins was confirmed via the identification of previously characterized FLCN interactors, such as FLCN interacting protein 1 (FNIP1) [31] and FNIP2 [32], as well as PKP4 [13, 14] (Fig. 4B) and the BRCA1 A complex component, BRE [33]. When considering identified interactors where more than one unique peptide was identified, 541 potential FLCN binding proteins were mapped by DAVID analysis into functional clusters (Fig. 4C). This revealed that the strongest enrichment was in proteins involved in translation, in keeping with the known function of FLCN in the mTORC1 pathway. Of interest, we identified protein folding chaperones as highly enriched in our dataset, including all 8 components of the TRiC/CCT chaperonin complex and two isoforms of HSP90, plus the co-chaperone CDC37. The chaperonin CCT helps fold both mLST8 and Raptor, part of mTORC1 [34], while the R2TP complex (which includes RUVBL1 and RUVBL2), together with heat shock protein 90 (HSP90), is a chaperone for the assembly of protein complexes including phosphatidylinositol 3-kinase (PI3K)-like kinases (PIKKs) such as TOR and PRKDC, more commonly called DNA-dependent protein kinase (DNA-PK) (highlighted in Fig. 4B and 4D) [35]. Additionally, FLCN was previously described as a HSP90 client protein, where the HSP90-FLCN interaction enhances the stability of FLCN [36]. Several CCT substrates are cell cycle proteins, particularly at the G_1_/S phase (discussed in [37]), so it was interesting to observe that cell division was also a top 10 enriched process (Fig. 4C). Comparing different stages of the cell cycle, there was a higher proportion of FLCN-binding proteins linked to the G_1_/S phase (Supplementary Fig. 3A), including CDK1, CDC20, TP53 and the RB binding protein 7 (RBBP-7). This analysis supports our RNA sequencing findings, indicating that FLCN is linked to the G_1_/S cell cycle. One cell cycle protein identified, DNA-PK, was our top interactor, with the most peptides identified (Supplementary Fig. 3B). This protein also has a role in the DNA repair, another enriched process amongst our interactome (Fig. 4C). Indeed, a number of interactors had involvement in metabolism as well as cell cycle and DNA damage (Fig. 4D).

### DNA-PK interacts with FLCN

**Figure 5:**
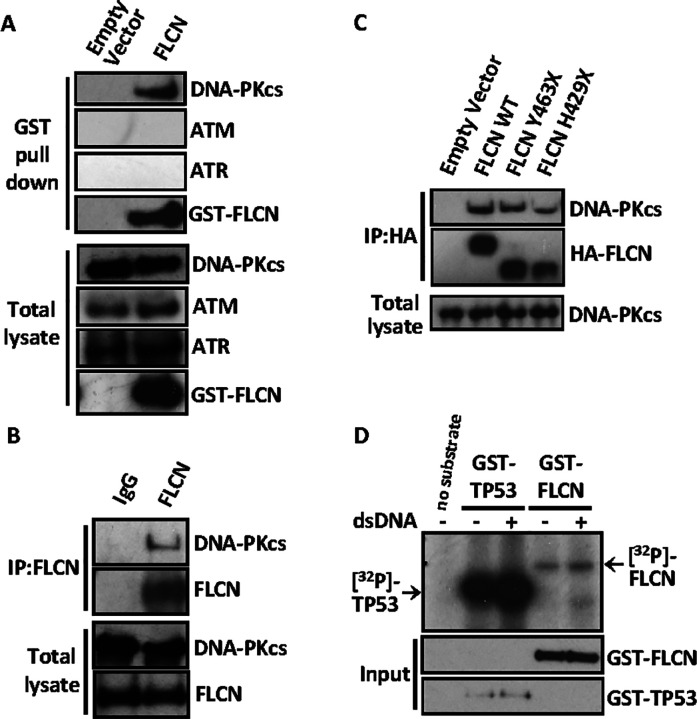
FLCN interacts with DNA-PK. **(A)** GST-tagged FLCN was overexpressed in HEK293 cells and used as a bait protein to validate protein interactions between FLCN and endogenously expressed DNA damage components (DNA-PKcs, ATM, ATR) by GST-pull down. **(B)** Endogenous FLCN was immunoprecipitated using an antibody raised against N-terminal FLCN and the interaction of endogenous DNA-PKcs was detected by western blot. **(C)** HA-tagged FLCN constructs (wild type FLCN (WT), and two patient derived C-terminal truncated mutants (Y463X and H429X) were over-expressed in HEK293 cells, immunoprecipitated using anti-HA antibodies and bound endogenous DNA-PKcs was detected by western blot. **(D)** DNA-PK kinase assays were performed, using GST-FLCN or GST-TP53 that was overexpressed and purified from HEK293 cells. Incorporation of radiolabelled phosphate [^32^P] was determined with active DNA-PK that was further induced with supplementation of short double-strand DNA (dsDNA).

As our top inteactor, with 66 unique peptides and 17.9% coverage (Supplementary Figs. 3B and C), we focused our attention on DNA-PKcs. It plays an important role in cell cycle check point control during DNA damage, which correlates with our transcriptomic work that links *FLCN* knockdown to cell cycle dysregulation (Fig. 2B) and interaction network with FLCN (Fig. 4B–D). As there are limitations to over-expressed protein purification methods, i.e., potential non-specific/artefact protein binding to either beads or mislocalized over-expressed proteins, we carried out further FLCN-binding validation experiments on DNA-PKcs. We initially examined whether endogenous DNA-PKcs co-purified with GST-tagged FLCN (Fig. 5A). We observed robust interaction of DNA-PKcs with GST-FLCN and saw no DNA-PKcs in the empty vector control, showing no non-specific/aretfact binding of DNA-PKcs to beads. Other PIKK family members, ATM or ATR, did not immunoprecipitate with FLCN. Further validating the DNA-PKcs interaction, we observed interaction of endogenous DNA-PKcs with immunoprecipitated endogenous FLCN (Fig. 5B). Next, we compared association of DNA-PKcs with wild-type FLCN and BHD patient-derived mutants, Y463X and H429X (Fig. 5C). Both C-terminal truncation FLCN mutants showed interaction with endogenous DNA-PKcs. We then considered that FLCN might be a direct substrate of DNA-PKcs. Therefore, *in vitro* DNA-PK kinase assays were performed, using TP53 as a DNA-PK substrate control (Fig. 5D). Supplementation of dsDNA was used to further enhance DNA-PK’s kinase activity. Unlike TP53, where DNA-PK-mediated phosphorylation was enhanced with supplementation of dsDNA, we observed a much weaker level of [^32^P]-incorporation into FLCN. Inclusion of FNIP1 or FNIP2 did not further enhance this low level of phosphorylation (data not shown). Given the low levels of [^32^P]-incorporation into FLCN that was not further enhanced with supplementation of dsDNA, FLCN is unlikely a direct substrate of DNA-PK in these assays.

### Cell cycle progression is dysregulated following long-term loss of FLCN

**Figure 6:**
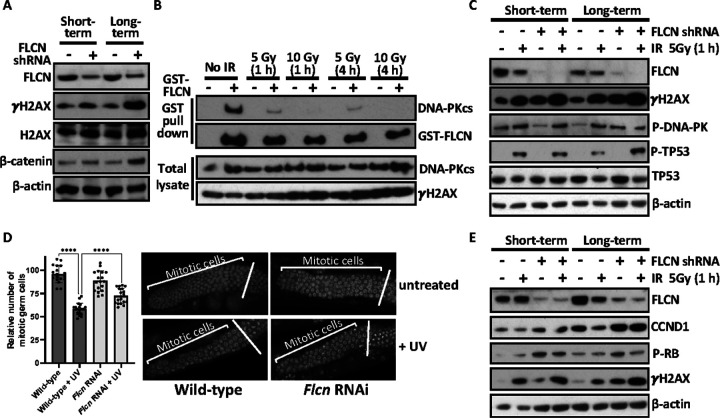
*FLCN* knockdown promotes cell cycle progression. **(A)** Serine 139 phosphorylation of histone variant H2AX (γH2AX) was assessed under basal conditions in HK2 cells with and without short-term or long-term *FLCN* shRNA knockdown (non-target shRNA was used as a control). (**B**) FLCN/DNA-PKcs interaction was investigated following IR induced DNA damage. GST-tagged FLCN was overexpressed in HEK293 cells that was then subjected to IR (5 or 10 Gy) and left for either 1 or 4 h prior to cellular lysis, as indicated. GST-FLCN was purified and endogenous DNA-PKcs detected by western blot. (**C**) In the HK2 cells with and without short- or long-term *FLCN* shRNA knockdown, markers of DNA damage were assessed following 5 Gy IR for 1 h, where indicated. γH2AX, P-DNA-PKcs (Ser2056) and the downstream DNA-PK substrate, P-TP53 (Ser15) are shown, alongside. FLCN and b-actin as controls. (**D**) Mitotic germ cells were quantified in *C. elegans* with and without *Flcn* siRNA knockdown and subjected to UV damage, where indicated. Mitotic germ cells were scored are graphed. Representative images of the mitotic cells are presented, (*n*=18). (**E**) Same lysates from panel C were analysed for G_1_/S phase cell cycle markers, CCND1, and P-RB1, with FLCN, γH2AX and b-actin as controls.

As DNA-PKcs regulates the phosphorylation of H2AX in response to cell cycle progression and DNA damage [38], we next analysed γH2AX (H2AX phosphorylated at Ser139). Following short-term *FLCN* knockdown γH2AX was enhanced and long-term *FLCN* knockdown elevated γH2AX further (Fig. 6A). γH2AX is classically regarded as a marker of DNA damage involved in the surveillance and repair of double strand breaks, such as those induced by ionising radiation (IR). However, γH2AX has also been reported to occur independently of double strand DNA breaks [39], in mitotic cells [38] and in response to serum starvation [40]. Therefore, we next assessed the interaction of DNA-PKcs and GST-FLCN following IR and found that endogenous DNA-PKcs dissociated from GST-FLCN after IR treatment at 5 and 10 Gy (Fig. 6B). This reveals that FLCN/DNA-PK association is regulated by DNA damage. 5 Gy IR treated cells were analyzed for markers of DNA damage to determine whether *FLCN* knockdown altered DDR signaling. IR enhanced γH2AX as expected, with a higher basal and IR-induced level of γH2AX in the FLCN-deficient HK2 cells (Fig. 6C). DNA-PKcs autophosphorylation is essential for the appropriate regulation of DNA strand end processing, enzyme inactivation, and complex dissociation from DNA (see review [41]). However, no change in DNA-PK autophosphorylation was observed upon *FLCN* knockdown. While phosphorylation of TP53 at Ser15 was induced under IR, there was no difference when comparing *FLCN* knockdown to wildtype (Fig. 6C). This data shows that although γH2AX is elevated following *FLCN* loss, *FLCN* loss has no direct impact on IR induced DNA-PK signaling, i.e., elevated γH2AX is unlikely to be reflective of double strand DNA breaks. When the RNA sequencing data with and without *FLCN* knockdown was run through Mutect2, there was no evidence of enhanced DNA mutations (data not shown). This indicates that *FLCN* loss is unlikely to be enhancing DNA damage, but might be more related to cell cycle control linked to DNA-PK.

To examine FLCN’s role in an alternative system, we analysed *C. elegans* with and without *Flcn* RNAi knockdown. *C elegans* has previously been used as a model organism to characterise the effects of DNA damage and cell cycle check point control in germ cells (reviewed in [42]). While we observed no change in the relative number of mitotic germ cells upon *Flcn* knockdown, *Flcn* knockdown worms were defective in cell cycle arrest by UV-induced DNA damage. We observed by a small increase in the relative number of mitotic germ cells after UV (Fig. 6D). This supports a hypothesis that *Flcn* knockdown results in dysregulation of cell cycle control following DNA damage. To determine whether cell cycle arrest defects following FLCN loss is also linked to the G_1_/S phase transition in mammalian cells, we examined HK2 cells for CCND1 expression and RB1 phosphorylation following IR treatment (Fig. 6E). CCND1 expression was basally higher after *FLCN* knockdown, in line with previous work [16]. Upon long-term *FLCN* knockdown, CCND1 expression was further increased (Fig. 6E). In healthy cells, CCND1 protein levels are typically reduced after IR, as part of a normal DNA damage cell cycle checkpoint control mechanism [43]. However, in *FLCN* knockdown cells following IR treatment, CCND1 protein levels remained elevated, indicating a defect in the normal control of CCND1 after DNA damage. Increased RB1 phosphoryation was also observed after *FLCN* knockdown, implying an elevated level of active G_1_/S cyclin-CDK complexes. RB1 phosphorylation leads to E2F activation and entry into S phase, suggesting that knockdown of FLCN favours G_1_/S checkpoint slippage. Supporting this observation, transcriptomic analysis showed enrichment of E2F regulated genes in *FLCN* knockdown HK2 cells (Fig. 2B). Overall, *FLCN* knockdown leads to cell cycle dysregulation with and without DNA damage, where we observe heightened levels of CCND1 and RB1 phosphorylation as well as enhanced E2F-mediated transcription of S-phase genes.

## Discussion

RCC takes several decades to develop in BHD patients, despite their germline FLCN mutation, but most studies to date only analyze the immediate impact of FLCN loss in cells. Therefore, we explored the long-term consequences of *FLCN* loss through transcriptomic and proteomic analysis to better characterize FLCN as a tumor suppressor, and in doing so uncovered links to DNA-PK and cell cycle control.

Proteomic analysis revealed FLCN-interactors involved in protein folding, cell cycle and DNA repair, including DNA-PK. DNA-PKcs showed avid binding to both wild-type and BHD-patient derived FLCN mutants. Arguing against a non-specific binding artefact, this interaction was completely reversed under IR. DNA-PK has a key role in the DNA damage response. *FLCN* loss was previously linked to DNA damage, where FLCN-deficient cells were shown to be sensitive to PARP inhibitors due to impairment of BRCA1 A complex associated DNA repair ability [33]. In our study, we detected the BRCA1 A complex member, BRE, in our FLCN pulldown experiments, and also saw that long-term *FLCN* knockdown resulted in an elevated level of γH2AX. We believe this enhanced level of γH2AX is unlikely due to DNA damage, as other markers of DNA repair signaling were unaltered. DNA-PK has roles beyond the DNA damage response, including in cell cycle progression [38], so we propose that *FLCN* loss causes defects to cell cycle control. It is likely that the higher levels of γH2AX after *FLCN* knockdown reflects a known role of DNA-PK to enhance γH2AX expression during cell cycle control, which can occur independently of DNA damage [44]. Therefore, the large transcriptional changes in cell cycle genes observed upon *FLCN* knockdown are likely due to epigenetic alterations or altered gene expression downstream of metabolic signaling changes rather than an accumulation of unrepaired DNA errors.

While our study specifically focused on the G_1_/S phase of the cell cycle, it should be noted that FLCN has also been linked to the G_2_/M phase [45]. Upon *FLCN* knock down, we observed aberrant protein levels of CCND1. Previous work revealed that the translation of *CCND1* mRNA was negatively regulated by a FLCN regulatory cis-acting element in its 3’-untranslated region [16], which partially helps to explain why CCND1 protein levels become elevated upon FLCN-deficiency. We observe that CCND1 protein levels stay elevated even after IR induced DNA damage in the absence of FLCN, suggesting that the DNA damage cell cycle check point control may be compromised with FLCN knockdown. As RB1 phosphorylation was elevated upon *FLCN* knockdown, there must be a higher level of CDK activity towards RB1 in these cells. Analysis of the RNA sequencing provided further evidence of RB1/E2F activation, as E2F target genes were more highly expressed in the absence of *FLCN*. Furthermore, upon *FLCN* knockdown, we observed a modest reduction in the G_1_ population of cells, inferring potential increased proliferative drive through G_1_/S in the absence of *FLCN*. The high protein levels of CCND1 and RB1 hyperphosphorylation in long-term *FLCN* knockdown HK2 tumor spheroids likely contributes to their observed tumorigenicity.

Our interactome data and the enrichment of protein folding chaperones is consistent with previous observations that FLCN is folded and assembled into large multiprotein complexes. Recent publications have shown that FLCN is involved in megacomplexes controlling metabolism, such as the FLCN-FNIP2-Rag-Ragulator complex [46], the mTORC1−TFEB−Rag−Ragulator megacomplex, which includes the FLCN-regulated RagC [47].

By utilizing BHD cell models, we revealed that FLCN is connected to cell cycle control. The DDR pathway is defined through several key features, one of which is cell cycle checkpoint control that is regulated by DNA-PK. This helps prevent propagation of somatic mutations into progeny cells when DNA damage is present and not repaired. Two pieces of evidence indicates a defect in proper cell cycle checkpoint control upon FLCN loss. Firstly, *C.elegan* animals with *Flcn* knockdown had more mitotic germ cells following DNA damage compared to non-target knockdown. Secondly, in mammalian HK2 cells, CCND1/RB1-P remained elevated in the presence of DNA damage when FLCN was knocked down, while this checkpoint was engaged normally in wild-type cells (as observed by reduced CCND1/RB1 phoshorylation). Such dysregulation of cell cycle processes and metabolism could potentially cultivate more genetic lesions over time and thus drive the earlier onset of cancer progression seen in BHD patients.

## Data Availability

All datasets generated or analysed during this study are included in this published article and its supplementary information files. Data analysed during the current study are available from the corresponding author on reasonable request.
